# Imaging of Cardiac Valves by Computed Tomography

**DOI:** 10.1155/2013/270579

**Published:** 2013-12-29

**Authors:** Gudrun Feuchtner

**Affiliations:** Department of Radiology, Innsbruck Medical University, 6020 Innsbruck, Austria

## Abstract

This paper describes “how to” examine cardiac valves with computed tomography, the normal, diseased valves, and prosthetic valves. A review of current scientific literature is provided. Firstly, technical basics, “how to” perform and optimize a multislice CT scan and “how to” interpret valves on CT images are outlined. Then, diagnostic imaging of the entire spectrum of specific valvular disease by CT, including prosthetic heart valves, is highlighted. The last part gives a guide “how to” use CT for planning of transcatheter aortic valve implantation (TAVI), an emerging effective treatment option for patients with severe aortic stenosis. A special focus is placed on clinical applications of cardiac CT in the context of valvular disease.

## 1. Introduction

Multislice computed tomography (CT) is a new modality for noninvasive evaluation of cardiac valves, with new clinical applications arising over the past years. While multislice CT has been established for assessment of coronary arteries for a decade, the cardiac valves were neglected initially. One reason was the fact that echocardiography is a strong modality in clinical practice. Still, it has its own limitations, such as being related to observer variability and high individual differences in image quality pending on body habitus, or its flow dependency. Further, echocardiography has limitations in assessing valvular morphology. Therefore, multimodality imaging, including CT is required, for diagnostic workup of valvular disease.

The first part of this paper describes technical basics of CT, how to perform and optimize a multislie CT scan. Then, “how to” interpret valves on CT and how to diagnose the specific valvular disease, including prosthetic valve dysfunction, are illustrated. Finally, a guide “how to” utilize CT in patients with severe aortic stenosis scheduled for planning of transcatheter aortic valve implantation (TAVI) is provided. TAVI is an emerging treatment option in those populations.

Current scientific evidence and literature are reviewed. A special focus is placed on the most recent and useful clinical applications in terms of “when and why” we use cardiac CT in the context of valvular disease effectively in practice.

## 2. Technical Basics

*From Dream to Reality*. While in early 2000, 4D-cine imaging (“cine imaging”) of cardiac function by multislice computed tomography (CT) was a “dream not yet true”, continunous advances in mainly temporal, but also spatial resolution, have created new horizons. Since 2005, with 16, 64, or more slices and increasing gantry rotation speed, both spatial and temporal resolution were improved, respectively. The highest temporal resolution of 75 ms is currently achieved with second generation dual source CT, allowing the highest image quality with regard to moving structures such as cardiac valves. Notably, both left and right ventricular function can be quantified (ejection Fraction, volumes, etc.). It is no longer a dream, but true.

In order to assess valvular function by multislice CT, the acquisition of a CT dataset during multiple entire cardiac cycle is necessary. There are 2 different ECG-gating techniques available. First, retrospective ECG gating, in spiral mode, is the technique of first choice. During 5–10 RR-intervalls, the heart is captured with a low pitch of 0.2–0.5. Second, prospective dual step ECG-triggering has been introduced, a sequential scan technique. Hereby, the table moves “step by step” and covers in heart in 4-5 heart beats. Two padding (“pulsing”) windows are placed: one into diastole at full tube current, for imaging of coronary arteries. A second padding (pulsing) window covers the entire RR interval at 20% reduced tube mA, enables assessment of valvular function. Multiphase datasets are typically reconstructed at 5 or 10% intervals during the entire cardiac cycle. The advantage of prospective ECG triggering is a reduced radiation dose of mean 3.8 mSv [1], as compared to helical retrospective ECG gating. However, a regular heart rate is required in order to avoid misalignment artifacts [1].

Notably, very new low-radiation dose CT techniques such as high-pitch coronary CTA [2–4] with ECG-synchronization are only capturing the diastolic phase but allow for ultralow radiation exposure of only 1 mSv. Hence, valvular assessment for morphology, but not function, is feasible.

Cardiac arrhythmias such as atrial fibrillation or extrasystole are common in patients with valvular disease may result in deterioration of image quality. Inconsistent RR intervals lead to “misregistration” artifacts such as stair steps or “blurred hazy” images. ECG editing is an effective technique to trade off artifacts: “outlier” heartbeats (e.g., the extrasystolic beat) are “disabled” (=removed). On the other hand, if the interval between heart beats is too long, data loss occurs, and an additional 2nd image reconstruction window must be added.

It is generally not advised to examine patients with atrial fibrillation using 16- or 64-slice CT, because diagnostic quality is mostly not achieved in these patients. In contrast, the superior temporal resolution of dual source CT allows sufficient image quality in most patients with atrial fibrillation [5] and with high heart rates. Beta blockers should be used to lower and regulate heart rate (if not contraindicated, e.g., such as in patients with aortic stenosis).

*Iodine Contrast Media Injection.* In order to optimize chamber attenuation, timing of the contrast bolus is highly important. If injecting a monophasic bolus injection triggered into the arterial phase, as commonly performed for coronary CT angiography (e.g., with high flow of 5 cc/s), the right chambers are typically “washed out” (without any contrast), and both tricuspid and pulmonary valve are nonevaluable. In contrast, a biphasic contrast agent injection protocol is favorable over a monophasic injection protocol, to obtain right ventricular enhancement, which allows evaluation of all four cardiac valves and chambers, including the opportunity of regional and global left and right ventricular function analysis. For that protocol, first, a bolus with high flow rate (5 cc/s) is injected, followed by a second bolus with a lower flow rate of about 3.5 cc/s [6] The total volume of the bolus is split as follows: 80% high flow/20% low flow. Following the contrast bolus, about 30–40 cc of saline chaser should be followed, in order to ensure fast bolus transit and to optimize bolus geometry. Iodine concentration of the contrast medium is recommended to be high (>300 mg/dL). A further advantage of biphasic injection is the reduction of streak artifacts resulting from high contrast agent injection flow [6].

## 3. “How to” Evaluate Cardiac Valves by CT

*Multiplanar reformations (MPR)* on advanced 3-D post-processing workstations or thin client server based solutions are mandatory for aortic valve assessment. The aortic valve should be reviewed on thin slices MPR (1 mm slice width) and reconstructed in three different views (Figure 2) (left sagittal oblique, left coronal oblique, and cross-sectional oblique view of the valve (Figures 2 and 3). For mitral valve imaging, reconstruction of 4-chamber, 3-chamber, and 2-chamber views as well as short axis views of the mitral valve (Figure 4), is recommended.

**Figure 1:**
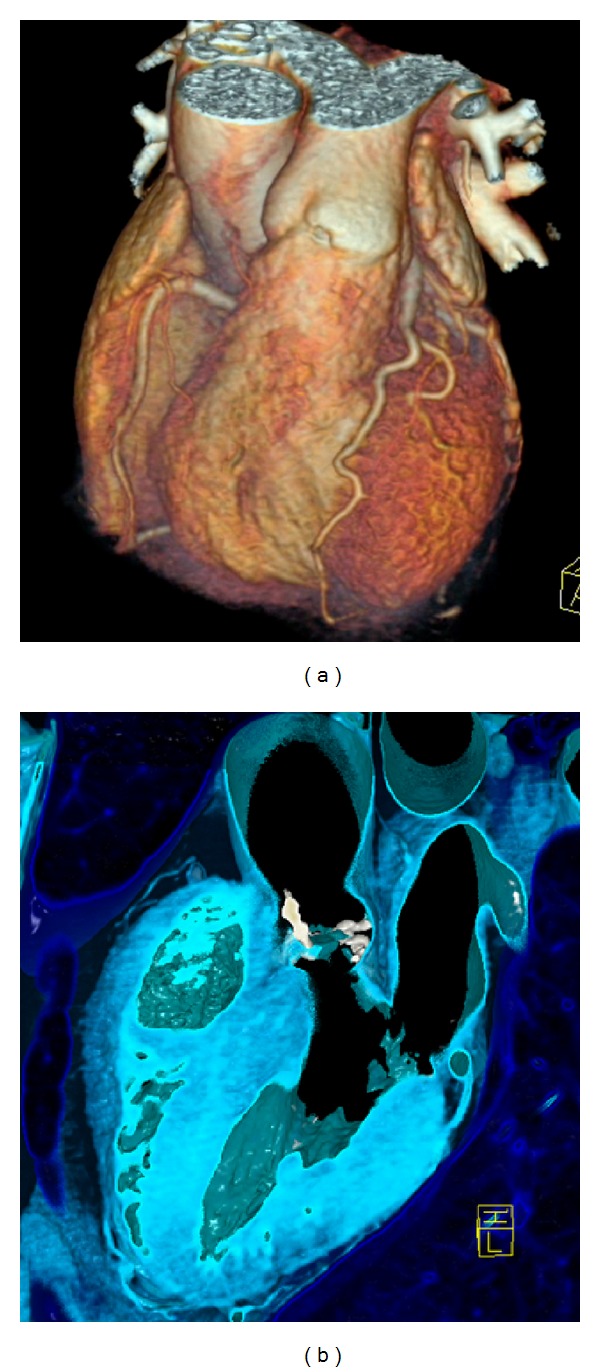
(a) *3-D VRT image of the heart by computed tomography*. The coronary arteries (LAD—left anterior descending—in the front) are shown. Cardiac valve evaluation from same CT data set is possible. (see pulmonary valve in the front). (b) *3-D image of the aortic valve. Severe calcification (white color)* of the aortic valve are pathognomonic for degenerative aortic stenosis (here shown in 3-chamber view). Further, quantification and characterization of aortic valve and annulus calcium predict complications during transcatheter aortic valve implantation (TAVI) such as postsurgery paravalvular regurgitation or annulus rupture during stent expansion.

*3-D Volume Rendering Technique (VRT)* allows true 3-D display of valvular surfaces and calcifications (Figure 1(b)), also for prosthesis (Figure 5). 

*Maximal intensity projections (MIP)* may be used to show morphology, while mostly MPR provide more accurate results. MIP however is a more appropriate postprocessing tool for the vascular tree (Figure 6). 

## 4. The Heart Valves and Its Diseases

### 4.1. Aortic Valve

The following paragraphs describe the utilization of CT for diagnosis of aortic valve morphology and dysfunction.

### 4.2. Bicuspid Valve

**Figure 2:**
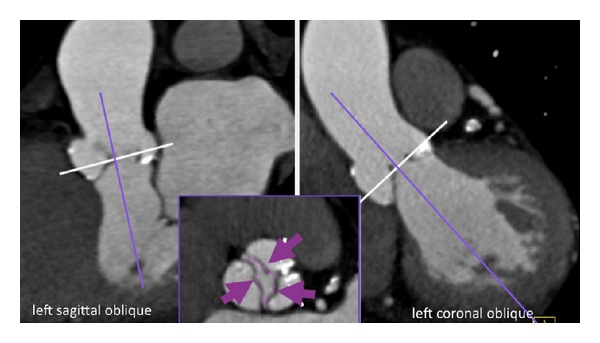
*Aortic stenosis: Aortic valve area (AVA) sizing by CT*. Using 3-multiplanar reformations (MPR), from left sagittal oblique (left) and left coronal oblique (right) views, a cross-sectional view of the aortic valve is generated (*lower mid image*). The white line indicates the plane of the cross-sectional axial oblique view in the low midimage inlay, which allows quantification of the inner aortic valve orifice area (AVA) (pink arrows). This area is traced by a digital caliper and reflects a marker for the severity of aortic stenosis (<1 cm^2^: severe critical). Valve morphology was bicuspid (fused raphe).

The diagnosis of a bicuspid valve by echocardiography [7, 8] is often challenging by transthoracic echocardiography, particularly if image quality is suboptimal due to patient habitus. In those patients, CT is a helpful troubleshooter: CT is accurate with a sensitivity of 94% and specificity of 100% [7] for detection of bicuspid valve shape. Both systolic and diastolic phases have to be reviewed and evaluated carefully. A raphe (in 85%) (Figure 2) or no raphe (in 15%), respectively, [7] is typically found by CT. A raphe indicated the fusion line of 2 leaflets (e.g., the left and right coronary). During diastole, bicuspid valves show the “linear sign” and, during systole, a “round” or “fish-mouth” opening orifice, respectively (Figure 2).

In rare cases, a “quadricuspid” aortic valve [9, 10] is present, consisting of four leaflets with an estimated incidence of 0.003 to 0.043%.

Diagnosis of bicuspid valve morphology is important for planning of patient management, particularly cardiac surgery, because the surgical technique may be modified. Beyond, bicuspid valves are prone to degenerate and develop dysfunction (both stenosis or regurgitation) earlier with age, hence being associated with worse prognosis requiring careful patient monitoring.

### 4.3. Aortic Stenosis

The systolic aortic valve orifice area (AVA) (Figure 2) is the parameter of choice for establishing diagnosis of aortic stenosis. AVA quantification by CT during the midsystole (5%–25% of RR-interval) is feasible [11–27] and proven accurate with *r* values of mean 0.8–0.9 as compared to transthoracic echocardiography. In more than 600 patients, a mean *r* value of 0.89 is calculated (Table 1) when comparing AVA sizing by CT with TTE. Two studies have compared CT with invasive angiography (Gorlin Formula) and found similar results (*R* = 0.9 and *r* = 0.91) in terms of high correlation among each other. All studies found slight tendency of AVA overestimation by CT. This may be explained by different measurement technique: TTE “estimates” the AVA based on Doppler Continuity Equation, (VTI: Velocity Time integral) with respect to flow and LVOT size, assuming round LVOT shape. However, the LVOT is exccentric and not round. In contrast, CT provides direct “anatomical” sizing. In clinical practice, patients with low-flow-low gradient aortic stenosis, may benefit direct anatomic sizing, in order to define severity of aortic stenosis.

For most accurate results, the mid-systolic phase with the maximum opening during cycle, but the smallest orifice within the selected phase, should be chosen for reconstruction of the aortic valve and AVA sizing by CT. The newest scanner technology [28] allows adaptive sequential ECG triggering into mid-systolic phase, hence reducing radiation dose significantly.

### 4.4. Aortic Regurgitation

Aortic regurgitation (AR) is identified by CT during end-diastolic phase, because leaflet does not co-adapt fully. A central valvular “leakage” is pathognomonic and serves as diagnostic criterion for aortic regurgitation [29, 30]. Feuchtner et al. [29] have assessed quantification of the aortic regurgitant area by CT in 30 patients. Sizing the aortic regurgitant orifice area (ROA) was reliable for discrimination between severity degrees of AR with CT when ROA cut offs of <25 mm^2^ for mild and >70 mm^2^ for severe were set.

Alkadhi et al. [31] report about quantification of AR fraction and volume based of left and right stroke volume difference, in a comparative study in 53 patients with aortic regurgitation and 29 healthy controls. A high correlation of quantification of AR fraction and volume as compared to echocardiography was found. If AR volume cut-offs of <30 mL and >60 mL for mild, moderate, and severe AR were used, the sensitivity of CT was high with 93%.

### 4.5. Mitral Valve

Diagnostic imaging of the mitral valve is *and will* remain the domain of echocardiography, while mitral valve assessment by CT is feasible [31]. The mitral valve consists of an anterior and a posterior leaflet and anatomy as well as dynamics [31] using 4D imaging can be identified by CT. CT provides the opportunity of mitral orifice area sizing (MOA); however, its clinical use is limited [32]. In contrast, there are some useful clinical scenarios, in which, cardiac CT can be applied for mitral valve evaluation.

### 4.6. Mitral Valve Prolapse

In a multicenter study on 112 patients [33], the diagnostic performance of CT for assessing mitral valve prolapse was evaluated. 3- and 2-chamber views were the most reliable planes for identifying a patient with mitral valve prolapse. The accuracy of CT compared to transthoracic echocardiography was high with a sensitivity of 96%, a specificity of 93%, and a NPV of 96%. CT allowed differentiation of “flail” leaflet and “bowing” (= billowing) valve characteristics. Leaflets were regarded as thickened, if >2 mm in diameter, indicating myomatous, degenerative, or inflammatory disease.

### 4.7. Mitral Annular Calcification (MAC)

MAC mostly only involves the posterior mitral ring (j or U-shape) but may affect the entire ring (O-shape) (Figure 4). MAC may grow “mass-like” into the myocardium and mimic a tumor. Particularly if a inner “hypolucent” area is present, this imaging feature is pathognomonic for “caseous” MAC (inner liquidification). In suspected cases of mass-like MAC by echo, multimodality imaging including CT should be performed in order to confirm diagnosis, and in order to clearly distinguish a MAC from a tumor, CT is most helpful [34, 35].

### 4.8. Valvular Mass

**Figure 3:**
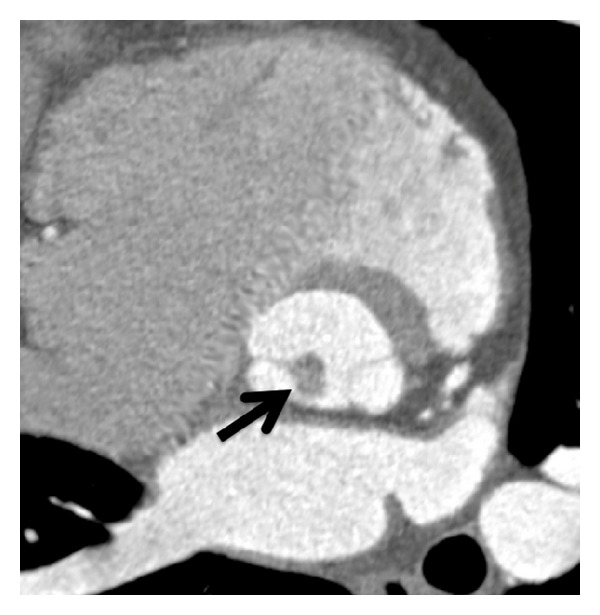
*Papillary fibroelastoma of the aortic valve: a round-shaped hypodense mass*. The mass is attached to the noncoronary cusp (black arrow). Cross-sectional view of the aortic root.

**Figure 4:**
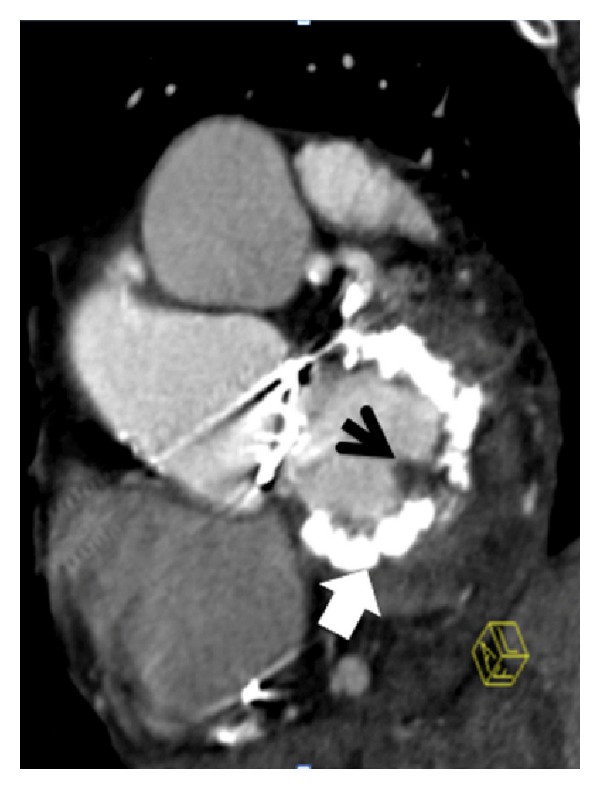
*Mitral annulus ring calcification (MAC) hyperintense (big white arrow)*. This patient also had infective endocarditis; therefore, a *mitral valve vegetation*—hypodense-black (round, mass like) left-sided (black arrow) was found, attached to the calcified mitral ring. Short axis view of the mitral valve.

Any unclear mass attached to a leaflet needs further diagnostic follow-up. In many cases, echocardiography does not fully clarify entity of a mass. For example, tumors, thrombi, vegetations, or even calcification appear hyperechogenic on echo and may not be differentiated from each other. In such cases, CT is a precious tool to differentiate hyperdense calcium from hypondense soft-tissue masses such as tumors, or thrombi, or vegetation. Further, iodine contrast uptake can be measured by HU (Hounsfield Units), which permits further differential diagnosis between vegetations (Figure 4), tumors such as papillary fibroelastoma (Figure 3), the most common heart valve tumors, or thrombi [36].

### 4.9. Mitral Valve Surgery

For planning minimal invasive cardiac surgery [37, 38] or mitral clip implantations, surgeons have recently raised awareness for the potential use of CT, in terms of evaluating leaflets calcium [37] or sizing the leaflets including measurement of tethering, respectively, such as for planning of mitral clip implementation via transcatheter route. The closeby anatomic relationship of the circumflex artery (CX) and the mitral annulus can be exactly measured by CT, and potential injury of the CX is prevented.

Finally, not at least, it must be mentioned that both the mitral and aortic valve are always “imaged” complementary on a normal coronary CT Angiogram exam. The spectrum of clinical indications for coronary CT-angiography is wide [36] and expanding still. Thus, it is the ethic and legal obligation of every radiologist or cardiologist, to report on any pathologies apparently present in the images. Hence, awareness of incidental, but relevant pathologies affecting cardiac valves, is of paramount importance for any radiologist reading coronary CT-angiography exams.

Further, in conclusion, the consensus document of ACCF/SCCT/ACR/AHA/ASE/ASNC/NASCI/SCAI/SCMR 2010 for appropriate use criteria for cardiac computed tomography [36] does recommend evaluation of native valves by CT and graded this indication as “appropriate”, if results from other modalities are not conclusive.

### 4.10. Infective Endocarditis (IE)

In a pilot study on 37 patients [34], a sensitivity of 97%, a specificity of 88% for diagnosis of infective endocarditis by CT was observed compared to TEE and/or the intraoperative specimen. A good correlation (kappa = 0.84) for detection of specific valvular lesions occurring in the context of infective endocarditis (IE) was noted. Valvular lesions in infective endocarditis included vegetations (Figure 4), which are usually mass-like, either longitudinal or round-shaped, without iodine contrast agent if new-acute, or with minimal uptake if older and vascularized. Overtime, vegetation may calcify. Further findings in the context of IE detected in this study [34] by CT were abscess, leaflet perforation or fistula between chambers, and/or great vessels.

Beyond, mobility of vegetations was diagnosed in 96% by applying 4-D cine imaging loops. While large perforations were detected, small leaflet perforation <2 mm size was missed. In clinical practice, patients with infective endocarditis scheduled for surgery often require a noninvasive coronary CT angiography to assess coronary artery disease status. Invasive coronary angiography via transcathether contrast injected poses the patient at high risk for embolization originating from mobile valvular lesion; thus it should be rather avoided and noninvasive coronary CT angiography used instead.

In conclusion, CT is particularly helpful for assessing extensive paravalvular involvement, including fistula and large abscess erroding the adjacent myocardium, the aortic root, or even extracardiac structures, and for precise surgery planning.

## 5. Prosthetic Heart Valves 

**Figure 5:**
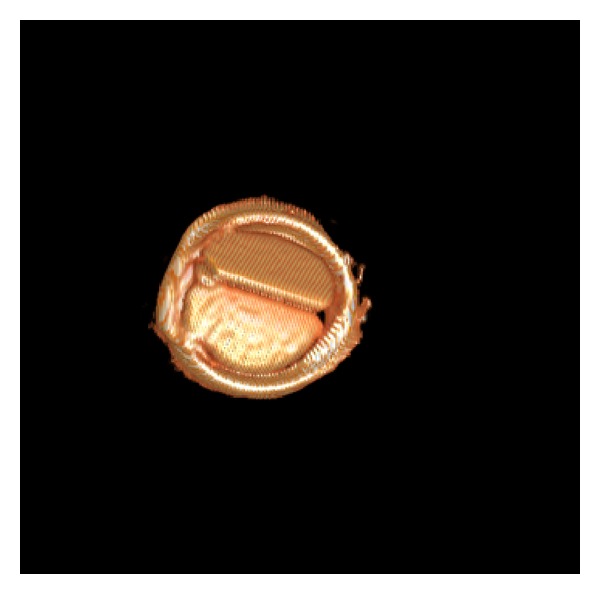
*Metallic mechanic prosthetic valve by CT*: 3-D VRT reconstructions permit the evaluation of leaflet dysfunction and valve obstruction. Here: St. Jude mechanic aortic valve, closed during diastole (normal finding). These valves cause artifacts of echo; hence, CT can act as troubleshooter in case of suspected dysfunction or infection.

There is striking scientific evidence that CT [39, 40] is a valuable modality for the evaluation of prosthetic heart valves and performs superior to established modalities such as transesophageal echocardiography (TEE) and fluoroscopy. This is explained by the fact that the CT allows both full 3D (Figure 5) and 4D-cine loop evaluation with less artifacts from metal compositions as compared to echocardiography.

Habets et al. [41] found in a pivotal study on 25 patients high correctness of CT (100%) compared to transthoracic echocardiography (TTE) for detection of pannus, prosthetic valve dysfunction, suture loosening (paravalvular leak), and pseudoaneurysm.

Tsai et al. [42] conducted a comparative study between CT, TEE, and surgery in 15 patients. The authors observed that MDCT findings compatible with valve obstruction were confirmed at surgery or autopsy in the majority of patients. The most common causes for obstruction were subprosthetic tissue (*n* = 6) and abnormal anatomic orientation of the device (*n* = 3). Multidetector CT also detected leaflet motion restriction in more patients as compared to fluoroscopy (*n* = 7 versus *n* = 4), out of those 5 were confirmed by surgery. Multidetector-row CT only missed one periprosthetic leak. The study concludes that this initial experience demonstrates that CT implies that detection of prosthetic valve obstruction by CT is helpful and improves diagnosis as compared to echocardiography or fluoroscopy.

Fagman et al. [43] included 27 patients before surgery. The correlation of CT with TEE was moderate for abscess (0.68) and high for aortic wall infection (0.83), dehiscence (0.75), and moderate for vegetations (0.55). The highest performance (0.88) was found if both TEE and CT were used combined to set up diagnosis of prosthetic valve infection.

Chenot et al. [44] noted in a study on 34 bioprosthetic valves that the AVA at CT correlated highly to effective orifice area (EOA) by TTE (*r* = 0.93, *P* < .001). In dysfunctional bioprosthesis (*n* = 34), CT results showed a variety of morphologic abnormalities suggesting structural valve deterioration (SVD), such as leaflet thickening, thrombotic material, or leaflet calcification (*n* = 1). Structural valve deterioration (SVD) is characterized by tissue degeneration, calcification, cusp tears, and increased stiffness, that my cause of device failure, stenosis or regurgitation.

Multidetector CT results demonstrated restriction of leaflet motion indicated by lower EOA (64 degrees +/−5 versus 79 degrees +/−3, *P* < .0001) in dysfunctional AVRs than in normal.

Pache et al. [45] recently reported successful detection of thrombosis by CT in a transcatheter prosthetic valve with a stent.

Comprehensive coronary artery disease assessment is a major advantage of CT in patients scheduled for surgery, who ofter need preoperative coronary artery stenosis grading [46, 47].

Symersky et al. [48] found in 89 patients with prosthetic heart valves that only 3.7% of coronary segments were nonevaluable. These patients had specific prosthetic devices with artifact enhancing material, such as Cobalt-chrome compositions, used, for example, for the Björk-Shiley and Sorin tilting disc valves. In contrast, more commonly used biological and titanium-containing valves such as the St. Jude bileaflet valve (image) did not cause artefacts hampering image quality of the coronary arteries, and all coronary segments were evaluable.

Not at least, coronary bypass graft patency [49] can be assessed before redoing surgery.

### 5.1. Prosthetic Heart Valve Infection

The diagnosis remains challenging, because the clinical presentation is often non-specific. Echocardiographic evaluation is often difficult due to artifacts from metallic leaflets. Several case reports indicate that CT evaluation is helpful, but adding 18-FDG-PET CT may even improve detection of abscess [50] or bioprosthetic valve infection [51].

Saby et al. [52] most recently published a landmark paper showing the added value of 18-FDG PET in setting up the diagnosis of prosthetic valve infection/endocarditis (PVE). In 72 patients with suspected PVE, 18-FDG PET increased the sensitivity for detection of PVE from 70% to 97%. The rate of “possible” but uncertain diagnosis was significantly reduced from 40 cases to 23 cases. In conclusion, using abnormal high FDG uptake is recommended as novel major criterion for diagnosis of PVE, according to Duke criteria.

### 5.2. Conclusion

As outlined in the consensus document of dedicated societies ACCF/SCCT/ACR/AHA/ASE/ASNC/NASCI/SCAI/SCMR 2010 for appropriate use criteria for cardiac computed tomography [36], CT is recommended for evaluation of prosthetic valves (*A* = appropriate indication), for further diagnostic follow-up after initial echo screening. The newest data indicate that 18-FDG PET results in improved accuracy for setting up diagnosis of prosthetic valve infection.

## 6. CT for Planning of Transcatheter Aortic Valve Implantation (TAVI)

Severe symptomatic aortic stenosis (AS) is common in the elderly and associated with high 1- and 5-year mortality of 40% and 68%, respectively [53]. Transcatheter aortic valve implantation (TAVI) has recently been established as viable alternative treatment option for inoperable and high-risk patients with symptomatic severe aortic stenosis over the past 10 years, and an expansion for use in intermediate-risk patients is currently discussed [54–57].

High success rate of 95%-96% success rate has been reported in one multicenter trial (PARTNERS). Another multicenter trial of 1038 patients enrolled at 32 centers (SOURCE-registry) showed as well excellent survival with 76% after 1 year [54]. Improved quality of life accounts for another major benefit of TAVI [57]. New 2-year outcome data [58] recently confirmed procedure success.

Multidetector computed tomography (CT) [59, 60] is playing a key role in patients scheduled for TAVI, because it is providing detailed morphological aortic valve and root assessment, aortic annulus sizing, in addition to the evaluation of the suitability of vascular access route (transfemoral versus transaortic versus transaxillary or transapical), and a prediction of the appropriate C-arm angulation/implantation plane.

The following review section focuses on preoperative CT assessment which is mandatory to select a patient for TAVI and to avoid intra- and postprocedural complications such as paravalvular regurgitation.

### 6.1. TAVI-Patient Selection

Still one of the most crucial aspects in TAVI planning is identifying the right patient. Considering that two thirds of all deaths after TAVI are non-cardiac, a multidisciplinary approach is of utmost importance to evaluate the potential gain of life quality while keeping preexisting comorbidities and possible complications in mind.

This is increasingly emphasized as current outcome prediction risk models are lacking important variables like frailty, liver disease, or the presence of a porcelain aorta.

Clinical factors help in the decision making process of patient eligibility for surgical aortic valve replacement (SAVR) or TAVI. Furthermore, transcatheter implantation feasibility can be effectively performed by CT by using following assessments.

### 6.2. Vascular Access Route

Access selection is an important part of the patients' eligibility. Careful selection based on CT evaluation has been shown to significantly reduce the major vascular complication rate such as iliac artery dissection or rupture. Different valve devices may be implanted retrograde via transfemoral (TF), subclavian axillary (TS) or more recently using an ascending aorta approach (TAO). Antegrade access is also possible via transapical insertion (TA) via the apex of the left chamber and minithoracotomy.

**Figure 6:**
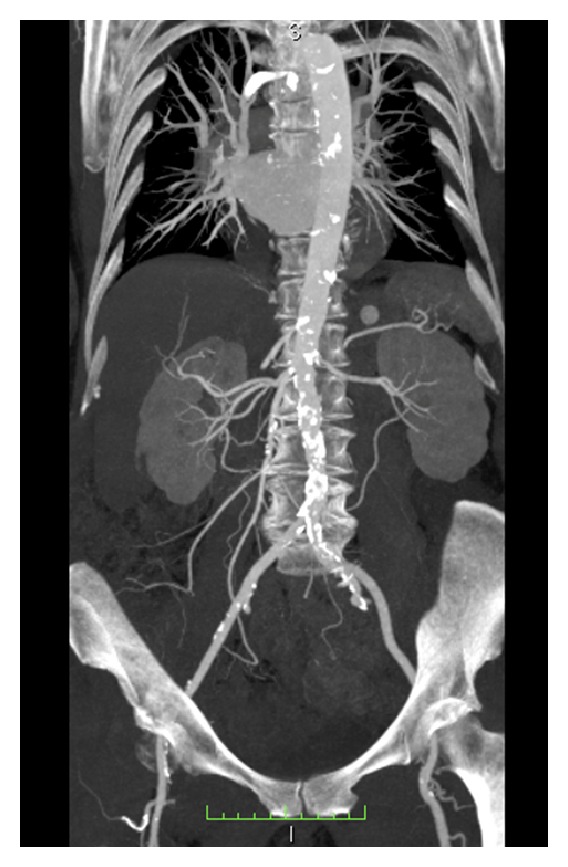
*Aortoiliac CT Angiography for planning of transcatheter aortic valve implantation (TAVI), using high-pitch 128-dual source CT*. Severe calcifications of the abdominal aorta but the right iliac artery are spared from atherosclerosis and do not show tortousity. Transfemoral access was possible via the right iliac artery.

CT angiograms (Figure 6) allow aortiliac vessel assessment and measurement of tortuosity, significant plaque burden or small vessel lumen. The transfemoral approach is the preferred retrograde delivery method, which is only it is possible if (1) tortuosity is less than 90 degrees in angulation, (2) iliac vessels are free of severe atherosclerosis, and (3) the minimal lumen diameter is appropriate for the delivery system of choice (pending on French size and vendor). Iliac vessels are sized along a centerline in 2 diameters, if the vessel is excentric (perpendicular), and the minimal diameter should be taken as effective, particularly if the vessel is calcified and no expansion of lumen can be expected. Over the past years, delivery systems have been decreasing in diameter, hence providing better features in patients with small vessels which permit a minimization of major vascular complications.

Moreover, the thoracic and abdominal aorta should not exhibit excessive calcifying plaque or high-risk soft atheromas Montgomery Class IV or V (Class IV: more than 4-mm in thickness, other CLASS V: high-risk features such as mobile, protruding lesions) or dissection flaps, due to increased risk of vascular complications as well as stroke or other arterial embolic events [61].

The major vascular complication rate was with 15.3% high in the PARTNERS trial, [62] but utilizing CT-Angiography restrictively in every patient [63] significantly reduced complication rate in a study on 137 patients from 2009 to 2010, major vascular complications decreased from 8% to 1% (*P* = 0.06), minor vascular complications decreased from 24% to 8% (*P* < 0.01), major bleeds from 14% to 1% (*P* < 0.01), and unplanned surgery from 28% to 2% (*P* < 0.01).

If the transfemoral route is not appropriate, the subclavian artery may be used (= transaxillary route). Again, the subclavian artery size must fit for the delivery system and it should be free from severe atherosclerosis.

Most recently, transaortal (via proximal ascending aorta) vascular access has been implemented in practice, with promising first results. For this approach, the ascending aorta anterior circumference must be free from atherosclerosis. Minimal plaque at the medial or posterior circumference may be appropriate.

### 6.3. Coronary Ostia Height Measurement

An important prerequisite for TAVI is an adequate height of the coronary ostia. Three-dimensional evaluation by CT is fundamental. Commonly, ostia heights greater than 12 mm from the annulus are generally considered safe. The most recent released data from a multicenter registry [64] implicate that a lower (>10.7 mm) cut-off is better in order to avoid ostia obstruction, resulting from either dislocation of valve material/calcium during expansion into the ostia and/or ostia overstenting. Although infrequent, with only 0.66% prevalence, a total of 44 patients suffered symptomatic coronary ostium obstruction following TAVI in a multicenter registry of 6,688 patients [64], but ostium was lower in those with CO with 10.7 mm versus 13.3 mm (OR: 2.17) who remained symptom-free. Low coronary ostia or long leaflets may lead to potential life-threatening overstenting, resulting in myocardial ischemia and infarct. Along with height measurements, the sinus of valsalva should be assessed. Shallow sinus may carry additional risk of coronary overstenting if combined with severe calcification or low ostia [64].

### 6.4. Aortic Valve and Root

*Morphology Assessment*. Another advantage of preprocedural CT is the evaluation of structural anomalies. Bicuspid valves are considered as relative contraindication for TAVI.

Additionally, structural assessments of the aortic root and left ventricular (LV) geometry including LV aneurysm, coronary artery disease (CAD), mobile thrombi, or structures or mitral regurgitation are routinely performed and important for TAVI planning.

### 6.5. Aortic Annulus Sizing and Its Implications for Prosthetic Heart Valve Sizing

**Figure 7:**
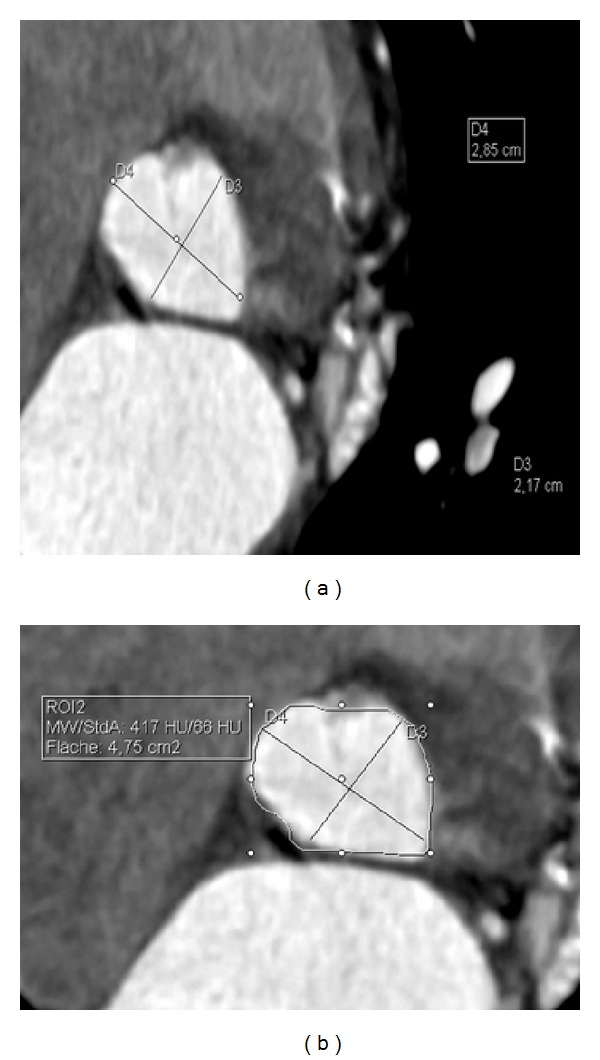
*Aortic annulus sizing by CT.* (a): The anterior-posterior (AP) (D3) and mediolateral diameter (ML) (D4) are measured on cross-sectional images. The mean of both diameters is commonly calculated for selecting the appropriate size of the transcatheter aortic valve prosthesis device finally used for TAVI (currently, 23 mm, 26 mm, or 29 mm devices). Exact fit is crucial to avoid paravalvular leakage and to ensure seamless adaption of the prosthesis with the aortic annulus. (b): Quantification of the annulus area is another valuable parameter for selection of the final valve size. Aortic annulus area is traced with a digital caliper (Area, 475 mm^2^).

While evaluation of iliofemoral vessels can be performed without ECG synchronization, it is obligatory to use aortic root and annular assessment to ensure accuracy [65]. Reconstruction slice width of 1.0 mm or less is required throughout the entire cardiac cycle. Traditionally, echocardiography has been the primary tool for aortic annulus measurements and consequently valve sizing. Due to its two-dimensional imaging approach, it is subject to important limitations regarding the assessment of the elliptical annulus. Messika-Zeitoun et al. have shown that the difference between long- and short-axis measurements (Figure 7) can have important clinical implication. Using different measurement methods in CT, the alternating mean diameter in CT would have influenced the TAVI strategy in 40% of patients [66]. Similar results have been reported by Wilson et al. and Gurvich et al. Both observed undersized valves (relative to CT measurements) in approximately 40% [67, 68]. They report appropriateness for larger devices in if CT was used instead of echocardiography for valve selection.

Hence, a three-dimensional approach such as that provided by CT (Figure 7) has shown benefits to prevent from over- or underestimation. Both the annulus diameters and the annulus area are valuable parameters. In the last years, MDCT has become the preferred imaging tool for preprocedural annular and root assessment, due to its high reproducibility and 3D nature.

References [69, 70] proposed guidelines show best results when using annular area or mean diameter for valve sizing. The highest correlation can be achieved if the measurements are performed in mid-systole in 25–35% of the heart cycle. Due to motion dynamics of the annulus, differences of up to 15% in size can be expected using different cardiac phases, given adequate imaging quality [65]. Both procedural reports and manufacturer's guidelines for self-expanding and balloon expandable prosthesis recommend oversizing of 10–15%.

Nonetheless, it should always be based on a multidisciplinary decision. Additional modifiers including left ventricular outflow tract (LVOT) calcification, shallow sinus of valsalva, or low left main coronary artery may affect valve selection.

### 6.6. A Postoperative Complication

*Paravalvular Aortic Regurgitation (PAR)*. Undersized prosthetic valves are the most important cause for paravalvular regurgitation (PAR), while excessive oversizing increases the risk of root injury. According to published results from the PARTNER trials, some degree of regurgitation can be observed in 80–96% (all grades, mostly trivial). Increased short-and long-term mortality correlated with PAR of grade mild or worse [54–56].

Several recent studies compared valve sizing using CT and echocardiography in correlation to postprocedural PAR. A single-center study from Jilaihawi et al. resulted in significantly less PAR (of grade mild or worse) when using CT for sizing [70]. Similarly, Hayashida et al. [71] published data from a single-center study that comprised 350 patients. They compared the incidence of PAR in transesophageal echocardiography (TEE) sized and CT-sized valves, with 175 patients undergoing TEE only and 175 both TEE and CT. Overall, 69 patients suffered from postprocedural paravalvular regurgitation of grade 2 or worse. Significantly less cases of PAR could be observed in the cohort with CT guided valve selection [71].

Investigating cases with oversizing, a recent study by Blanke et al. [72] evaluated potential causes of annular rupture. They followed 72 patients retrospectively after implantation of a balloon-expandable valve with pre- and postprocedural CT. Six patients were oversized >20%, three of them suffered from annular rupture. They also found significantly higher relative oversizing in patients with contained rupture compared to patients without contained rupture.

Thorough TAVI planning should also include quantification of aortic valve calcification. Nonenhanced CT can provide supplementary calcium scoring. Koos et al. reported significant association between total valvular calcium load and relevant PAR in 59 patients (they defined a threshold of 3000 Agatston score) [73]. Likewise, Haensig et al. found higher valvular calcification in patients with mild and moderate regurgitation compared to those without PAR [74]. Based on higher spatial resolution in contrast enhanced scans, Ewe et al. claimed a more accurate method for predicting PAR; rather than quantifying calcium, they found calcific lesions located at the aortic wall to correlate to postprocedural regurgitation [75].

This approach assigns greater importance to annular calcification than valvular calcium. It is believed that annular calcification interferes with optimal stent adaption, while calcium on the leaflet is pushed into the aortic wall further cranially. Similarly, John et al. suggested that the location of the lesions may be more important than the total calcium load [76].

### 6.7. Aortic Annulus Calcium and Prediction of Complications (Figure 1(b))

Barbanti et al. [77] very recently in May 2013 reported an increased risk (OR: 10.3) of annulus rupture during TAVI, if aortic annulus/LVOT calcium was graduated as moderate or severe (see Table 2) out of >100 patients from a multicenter registry, in which 31 had annulus rupture. Also, device oversizing >10% was a predictor of annulus rupture.

Feuchtner et al. [78] found in 94 patients after TAVI that specific aortic annulus calcium shape and size, are associated with increased risk of moderate to severe PAR. Protruding annular calcium (into the lumen) with >4 mm size and an increasing size were significant predictors of moderate to severe PAR (*c* = 0.7), while aortic leaflet calcium severity and asymmetry were not predictive. Beyond, adherent calcium to the wall had a “sealing” effect and prevents paravalvular leaks.

Most recently, Marwan et al. [79] observed in 105 consecutive patients that visual assessment of aortic annular calcification and the Agatston score correlated weakly but significantly with the degree of postprocedural AR (*r* = 0.31 and 0.24 and *P* = 0.001 and 0.013, resp.). Patients with moderate to severe PAR ≥ 2 also showed more severe calcification of the annulus but also higher aortic valve Agatston scores (1,517 versus 1,062, *P* = 0.005).

### 6.8. Prediction of Correct C-Arm Implantation Plane by CT: Optimization of Procedure Quality

Virtual prediction of the device landing zone has emerged as an increasingly accurate and useful application of CT for TAVI planning. TAVI procedures require hybrid operation rooms equipped with invasive angiography suites to implant the prosthetic valve under X-ray visualization. In order to guide implantation, a plane has been defined at the “deepest coronary sinus point,” with a projection of the connection points (“hinge-points”) of all 3 coronary cusp insertions into the aortic annulus (“3-coronary sinus alignment (3-CSA) plane”) in CT. With 3-CSA plane, it is possible to predict the intraoperatively used C-arm angulations for device implantation. The prediction of the optimal landing zone by CT leads to a reduction of contrast agent needed as well as the radiation exposure during implantation.

Gurvitch et al. [80]firstly described prediction of C-arm angulation by CT in 20 patients, with excellent or satisfactory projections in the majority (90% versus 65%, *P* = 0.06). The MSCT angle prediction was accurate but dependent on optimal image quality (optimal quality: 93% of predicted angles were excellent or satisfactory; suboptimal image quality: 73% were poor), highlighting the importance of high image quality and using the most advanced multilslice CT scanner technologies. For example, the 128 dual source high-pitch CTA (pitch, 3.2) [29] allows an optimal protocoll, with the advantage of a reduction of the effective radiation dose 10-fold as compared to a conventional spiral low-pitch multislice CT, with effective doses of 4.4 mSv for aortoiliac CT angiography [81]. Another benefit of 128-slice dual source CT is contrast agent volume decrease also due to shorter scan times of only 1-2 sec for a full body CTA [81, 82]. Only 40 cc was used by Wuest et al. [82] for a dedicated TAVI planning CT exam.

Other study groups found similar results. Kurra et al. [83] reported in 40 patients only a small difference between the caudal angulation in the RAO angiogram after matching CT images with the invasive angiogramm.

Plank et al. [84] showed in 49 patients a low left anterior oblique deviation between CT angiogram and the intraoperative C-arm projection plane finally used during invasive angiography, of 2.1 ± 2.7 degrees and a low craniocaudal deviation of 1.7 ± 3.0, respectively. Most importantly, contrast volume was reduced significantly from 81.8 to 59.4 mL (*P* = 0.05) when using 3-CSA plane estimation by CT for final intraoperative prosthesis implantation plane. Saving contrast volume is of special importance in this patient population, which is characterized by advanced age and a frequently impaired kidney function.

Binder et al. [85] compared 3-D rotational invasive angiogramm (IA) prediction and CT prediction of implantation planes, with a good correlation of both methods, concluding that both modalities are appropriate, while 3-D rotational IA had the disadvantage of adding +32 cc volume of iodine contrast.

Most recently, Arnold et al. [86] confirmed in a larger population of 75 consecutive patients significant less contrast agent volume injections for the entire TAVI procedure in patients with correct C-arm angle prediction as compared to those without it, (72 ± 36 mL versus 106 ± 39 mL, *P* = 0.001), due to a significant lower number of intraoperative angiogramms. Similar to the study by Plank et al. [84], deviation between CT and C-Arm was low with 3 ± 6 SD degrees [26]. CT predicted a suitable angulation (<5-degree deviation) in the majority of patients (84%) [26].

Intracardiac echocardiography [87] allows further contrast agent volume decline during implantation.

### 6.9. Conclusion

In patients with aortic stenosis scheduled for planning of aortic valve implantation (TAVI), CT is the modality of choice [88–100] and recommended by consensus documents of the SCCT (Society of Cardiac Computed Tomography Society) [88] as well as the Surgical Societies (AATS, ACCF, SCAI, and STS) [89]: firstly, to define optimal vascular access route, to select optimal prosthesis size based on annulus sizing [96, 97], but also to predict, and prevent intra- and postprocedural complications [98, 99].

## Figures and Tables

**Table 1:** Aortic valve area (AVA) sizing. Correlation of CT with echocardiography, fluoroscopy, and MRT.

	Patients (*N*)	Comparison of CT with:	Correlation coefficient (*R* value)
Ropers et al. 2009 [25]	50	TEE CATH	0.93 0.97
Lembcke et al. 2008 [16]	36	TTE TEE CATH	0.91 0.82 0.91
Li et al. 2009 [26]	36	TTE	0.79
Alkadhi et al. 2005 [31]	40	TTE	0.95
Feuchtner 2005	46	TTE	0.89
Bouvier et al. 2006 [21]	103	TTE	n/a; good agreement
Pouleur et al. 2007 [17]	48	TTE, TEE, MRT	0.92
Habis et al. 2007 [22]	52	TTE	0.76
Laissy et al. 2007 [23]	50	TTE	0.77
Leborgne et al. 2009 [19]	33	TTE	0.89
Feuchtner et al. 2007 [49]	36	TTE TEE	0.88 0.99
Tanaka et al. 2007 [20]	29	TTE	0.96
Lembcke et al. 2008 [16]	32	TTE CATH	0.86 0.90

Mean R value			**0.89**

TTE: transthoracic echocardiography; TEE: transesophageal echocardiography; CATH: invasive catheterized angiography (=fluoroscopy); MRT: Magnetic resonance tomography. *N*: count.

**Table 2:** Aortic annulus calcium classification of severity.

Moderate	Severe
>10% of annulus perimeter	>20% of annulus perimeter
1 nodule > 5 mm	1 nodule > 1 cm
2 nodules	Multiple nodules
